# Molecular Mechanisms Underlying Age-Related Ocular Diseases

**DOI:** 10.1155/2018/8476164

**Published:** 2018-05-21

**Authors:** Marialaura Amadio, Kai Kaarniranta, Heping Xu, Adrian Smedowski, Deborah A. Ferrington

**Affiliations:** ^1^Department of Drug Sciences, Section of Pharmacology, University of Pavia, Pavia, Italy; ^2^Department of Ophthalmology, University of Eastern Finland and Kuopio University Hospital, Kuopio, Finland; ^3^Department of Molecular Genetics, University of Lodz, Lodz, Poland; ^4^The Wellcome-Wolfson Institute of Experimental Medicine, School of Medicine, Dentistry and Biomedical Sciences, Queen's University Belfast, Belfast, UK; ^5^Chair and Department of Physiology, School of Medicine in Katowice, Medical University of Silesia, Katowice, Poland; ^6^Department of Ophthalmology, School of Medicine in Katowice, Medical University of Silesia, Katowice, Poland; ^7^Department of Ophthalmology and Visual Neurosciences, University of Minnesota, Minneapolis, MN, USA

The guest editors of this special issue are pleased to present a compendium of original research and review articles focusing on molecular mechanisms underlying age-related ocular diseases.

According to the World Health Organization (WHO), the 65-and-over global population is predicted to almost double over the next three decades. By 2050, less than one-fifth of the world's old population will reside in more developed countries; the functional visual decline associated with the elderly thus creates a challenge to be met by the national healthcare systems of industrialized countries. The WHO has identified some irreversible diseases, such as age-related macular degeneration (AMD), diabetic retinopathy, and glaucoma, as priority eye diseases in terms of prevention of visual impairment and blindness.

The lack of effective therapies for prevention or cure of such widespread sight-threatening diseases is mainly due to the insufficient understanding of the molecular and cellular events underlying the disease onset or progression. For this reason, basic scientific studies shedding light on the molecular and cellular responses to physiological/pathological stimuli (such as oxidative stress) are of great relevance and may help to translate pioneering “bench to bedside” research into effective clinical strategies.

After the awarding of 1974 Nobel Prize to Christian De Duve, who discovered the lysosomes and coined the term autophagy, and the awarding of 2016 Nobel Prize to Yoshinori Ohsumi for his discoveries of the mechanisms of autophagy, the importance of autophagy in physiological and pathological contexts has come to the forefront. The number of peer-reviewed publications with autophagy as a keyword has doubled in the last 5 years, at the present passing 32,500 papers. Accordingly, autophagy has been object of several contributions of this special issue.

Recent observations reveal that autophagy is declined in advanced AMD. J. Blasiak et al. wrote a review article for cellular senescence in AMD, the most common cause of irreversible blindness in the elderly of industrialized countries. Increased oxidative stress, protein aggregation, and inflammation are likely contributors to AMD pathology. Authors discussed cross talk of oxidative stress, DNA damage response, autophagy, and senescence in the retinal pigment epithelium (RPE) cell. Likewise, the article by L. F. Hernandez-Zimbron et al. provided a review of some AMD-related factors and mainly focused on feasible treatments targeting autophagy, oxidative stress, VEGF, and glial cell dysfunction, emphasizing the increasing relevance of stem cell-based research as a promising area in biology.

Despite significant breakthrough in the understanding of how autophagy modulates cellular processes, the entire puzzle of autophagy-related pathways is not yet completely elucidated. In particular, the role of posttranscriptional control of gene expression in autophagy is poorly characterized. Findings of the research article entitled “Autophagy stimulus promotes early HuR protein activation and p62/SQSTM1 protein synthesis in ARPE-19 cells by triggering Erk1/2, p38^MAPK^, and JNK kinase pathways” by N. Marchesi et al. focused on the early events occurring in response to a proautophagy stimulus and supported modulation of the autophagy-regulating kinases as a potential therapeutic target for AMD.

One of the factors promoting inflammation in AMD is lipid oxidation. Mao et al. provided *in vitro* and *in vivo* evidence that oxidized low-density lipoprotein (ox-LDL) promoted laser-induced choroidal neovascularization by increasing VEGF/PDGF and CYLD (tumor suppressor cylindromatosis) levels. The authors also proposed salvianolic acid A as a novel potential therapeutic reagent for exudative AMD in virtue of its beneficial antagonism on the ox-LDL-induced effects.

Many age-related ocular pathologies are multifactorial and are triggered by consecutive or overlapping insults that are hard to eliminate. The recovery of retinal health in spite of injury and the preservation (or reinforce) of its physiological defense thus represent a valuable strategy to fight factors that harm vision.

Several publications provide evidence that indicate a protective association between dietary antioxidant intake and the incidence of oxidative stress-related ocular diseases. D. Rajapakse et al. underlined the importance of physiological concentration of zinc for retina's homeostasis. In their *in vitro* study, the authors showed that zinc supplementation protects RPE cells from oxidative stress-induced death by improving mitochondrial function and preventing lysosome rupture.

The context of cytoprotection, of interest, is also the original *in vitro* study by S. Davinelli et al., showing that citicoline and homotaurine cotreatment protects primary cultured retinal cells from glutamate and high-glucose-induced neurotoxicity. Another contribution in the identification of molecules endowed with neuroprotective effects came from the *in vitro* study by Pittalà et al., who suggested the new nature-inspired NO-releasing compound VP10/39 as a promising candidate to protect RPE cells against oxidative stress, via induction of the Nrf2/Keap1/HO-1 pathway.

To this regard, one of the most comprehensive transcription systems to neutralize oxidative stress and maintain cellular homeostasis in the retina is the Keap1-Nrf2-ARE pathway, whose impairment has been associated with ageing. The review on the “Involvement of Nrf2 in Ocular Diseases” by S. Batliwala et al. provided thorough information regarding the relationship of Nrf2 and various age-related ocular pathologies, giving a perspective on possible therapeutic or preventive approaches targeting the Keap1-Nrf2-ARE pathway.

Novel cellular and animal models are needed to better understand the pathogenesis of age-related ocular diseases and to screen potential drugs. In this context, the original basic scientific study by A. Smedowski et al., on the FluoroGold-labeled organotypic retinal explant culture (FLOREC), proposed a new interesting and versatile ex vivo method to detect neurotoxicity/neurodegeneration in the retina and to test potential neuroprotective agents.

Among age-related conditions is the dry eye syndrome, which affects also young population and counts more than 300 million people worldwide suffering from this disease. The *in vivo* study by E. Y. Zernii et al. showed that the mitochondrial-targeted antioxidant SkQ1 is able to suppress oxidative stress and stimulate intrinsic antioxidant activity in the cornea and also improve the stability of the tear film.

Likewise, a contribution in the context of ocular surface inflammation came from the study entitled “Cyclodextrin enhances corneal tolerability and reduces ocular toxicity caused by diclofenac” by H. Abdelkader et al., investigating the use of cyclodextrins (CDs) to reduce ocular toxicity of NSAIDs, specifically diclofenac (Diclo). The authors provided *in vitro* and *in vivo* evidence that Diclo-*γ*-CD and Diclo-HP-*β*-CD inclusion complexes are able to reduce diclofenac-induced ocular toxicity and show fast healing rates without scar formation.

We hope the papers are informative for basic researchers, clinician scientists, and patients who are affected by age-related ocular conditions.

